# Cut-off points for low skeletal muscle mass in older adults: Colombia *versus* other populations

**DOI:** 10.12688/f1000research.109195.2

**Published:** 2023-06-27

**Authors:** Maria Camila Pineda-Zuluaga, Clara Helena González-Correa, Luz Elena Sepulveda-Gallego

**Affiliations:** 1 Department of Basic Sciences, Universidad de Caldas, Manizales, Research Group of Nutrition, Metabolism and Food Safety, Colombia; 2 Department of Public Health, Universidad de Caldas, Manizales, Research Group for Health Promotion and Disease Prevention, Colombia

**Keywords:** Cut-off points, skeletal muscle mass index, older adults, Colombia.

## Abstract

**Background:** The European Working Group on Sarcopenia in the Elderly defined sarcopenia as a geriatric syndrome with a diagnostic criteria of low skeletal muscle mass (LMM). Various sarcopenia consensuses recommend as cut-offs for LMM, the use of below 2 SDs from the mean skeletal muscle mass index (SMI) of a young reference group. Given the contrast between reported cut-offs, the objective of this study was to establish cut-offs for LMM from older adults in Manizales and compare them with those published in the literature.

**Methods:** This was a prospective, cross-sectional analytical study in 237 healthy elderly patients from the city of Manizales, Colombia. Anthropometric measurements of weight, height and body mass index were estimated. The SMI was estimated with the Xitron Technologies bioimpedance meter using the Janssen formula. For the comparison of SMI cut-offs, studies that evaluated this parameter with bioelectrical impedance analysis (BIA) were taken into account, in addition to being obtained from the −2 SD from the sex-specific mean of a young reference group.

**Results**: The cut-off points for SMI were 8.0 kg/m ^2^ for men and 6.1 kg/m ^2^ for women. There was a statistically significant difference when evaluating LMM from the cut-offs of the present study and those reported in Spain, Turkey, and Finland. The cut-off points of SMI derived from this sample of Colombian men and women may be adequate for the diagnosis in the Colombian geriatric population. However, we did not find significant differences when comparing the cut-offs for SMI from a population of older adults and young adults from the same city.

**Conclusions: **The cut-off points of SMI by BIA derived from a sample of Colombian men and women may be adequate for the diagnosis of LMM in the Colombian geriatric population or populations with similar characteristics to those of the sample evaluated here.

## 1. Introduction

Human aging is related to a set of modifications that produce irreversible alterations in body systems. ^1^ At the level of the musculoskeletal system, there is a reduction in muscle mass and a decrease in strength, which leads to the loss of functional capacity, which is known as sarcopenia. ^2^ The prevalence of this disease ranges between 15% and 50% and increases progressively with age depending on sociodemographic and gender variables. ^3^ Sarcopenia is one of the main causes of age-related disability ^4^ and is associated with frailty, muscle weakness, functional impairment, falls, fractures, dependency, institutionalization, and even premature death ^5^^,^^6^ it also imposes a significant economic burden on health services. ^7^ The European Working Group on Sarcopenia in Older People (EWGSOP) ^8^ defined this as a geriatric syndrome that requires the presence of low skeletal muscle mass (LMM) as one of its main diagnostic criteria. ^9^^,^^10^

Among the etiological mechanisms of LMM in older adults are the decrease in sex hormones, increased apoptosis, mitochondrial dysfunction, loss of motor neurons, decreased physical activity, endocrine alterations, among others. ^11^ That is how, LMM is a common problem for older adults around the world, with a prevalence ranging from 7% to 50%, ^2^ likewise, an annual decrease of 3% is reported after the sixth decade of life. ^12^

Estimation of skeletal muscle mass (SMM) can be done from various methods. In terms of precision and reproducibility, magnetic resonance imaging (MRI) and dual-X-ray absorptiometry (DXA) are ideal. ^13^^,^^14^ However, these methods are often expensive, complex, less available in clinical practice and commonly not used in studies with large sample sizes. ^15^ Bioelectrical impedance analysis (BIA) is not only the most widely available technique in low- and middle-income countries, ^3^ but also a reliable, portable, simple, inexpensive, and non-invasive method that estimates body composition and is taken as a valid substitute for total muscle mass with high correlation with MRI results. ^16^^,^^17^

To establish an LMM for older adults by BIA, the literature establishes some variables that must be considered, such as device, measurement methodology, BIA equation, and characteristics of the population studied, such as geographic location, age, race, sex, lifestyle, weight, height, among others. ^15^

Most researchers evaluate the total or appendicular SMM in young adults since in this age range the plateau of muscle growth is reached and subsequently remains relatively constant. ^2^ Similarly, the EWGSOP and the Asian Sarcopenia Group (AWGS) recommend as cut-off points for LMM, the use of minus two standard deviations (<−2 SD) from the sex-specific mean of a young reference group. ^18^^,^^19^ In contrast to the above, the cut-off points used can alter the interpretation of the results to a great extent. As an example, Baumgartner *et al*., ^20^ defined cut-off points for low muscle mass at 7.26 kg/m ^2^ and 5.45 kg/m ^2^, for men and women, respectively; while a previous study in our city, defined it as 8.39 kg/m ^2^ and 6.42 kg/m ^2^, for men and women. ^21^ These variations in the young population demonstrate the need to establish ethnic cut-off points and even those derived from older adults who live in the community to achieve real diagnoses that promote preventive and therapeutic strategies to impact sarcopenia.

Given the contrast between the data reported in the different geographic areas, the objective of this study was to compare cut-off points for LMM from older adults in Manizales with those found in the literature.

## 2. Methods

### 2.1 Study design and subjects

This was a prospective, cross-sectional analytical study. The calculation of the sample size was 195 people, as described by the Manizales City Council, which reported 40000 adults over 60 years of age in a city having approximately 400000 habitants and a sarcopenia prevalence of 15.5%, with 95% confidence and precision 5%. Finally, 237 older adults patients from the city of Manizales who live in the community were included in the study. The patients were evaluated between March 2019 and March 2020, in the University of Caldas health service provider. The inclusion criteria considered being over 60 years of age, being a resident of Manizales, not having a sarcopenia diagnosis, and being independent in carrying out activities of daily living. Once they attended the assessment site, it was confirmed that they could perform activities such as dressing, undressing, going up and downstairs, using the bathroom, control sphincters, and move from chair to stretcher. Exclusion criteria were considered when there were advanced or exacerbated chronic diseases, decompensated mental illnesses, partial or total amputation, pacemakers, wearing of non-removable metal parts or prostheses, moderate or severe disability, edema, and current use of diuretics were defined. This project was evaluated by the ethics committee of the Faculty of Health of the University of Caldas and was considered low risk (ethic’s board number CBCS-094), likewise, the participants signed the informed consent, before to being included in the study.

### 2.2 Anthropometric measurements

The measurements were performed in the morning, and it was confirmed that the patients met the standardization requirements for their evaluation. ^22^ Height without shoes was assessed with a Seca Heightronic-235 stadiometer ^®^, and the weight with light clothing with a PP2000 from Icob-Dectecto ^®^, with a scale of ±0.01 cm and ±0.1 kg, respectively. When there was a difference greater than 0.5 cm or 0.01 kg, a third measurement was taken, to have an average and record the final result. In addition, the body mass index (BMI) was established as weight over height squared, BMI = (weight/height ^2^).

### 2.3 Estimation of the SMI using BIA and cut-off points

For the BIA estimates, humidity and temperature were controlled using a dehumidifier (BFH416 from Bionaire TM), heater, and thermo-hygrometer (13307 from Delta Trak ^®^, ±0.1°C). BIA was performed three times on the dominant hemi-body on a non-conductive table with the Hydra 4200 Xitron Technologies bioimpedancemeter ^®^. The SMM was calculated from data at the resistance of 50 kHz and the predictive equation of Janssen *et al*., validated for the Hispanic population ( equation 1) ^23^:

$$\mathrm{SMM}\;\left(\mathrm{kg}\right)=\left[{\text{(height}}^{2}/R_{\text{50}}\;x\;0.401+\left(\text{gender}\;x\;3.825\right)+\left(\mathrm{age}\;x-0.071\right)\right]+5.102.$$ (1)

In this, the height is expressed in cm and the BIA resistance (R _50_) in ohms. For gender, 1 is for men and 0 to women, and age in years. Subsequently, to determine the SMI the SMM is normalized for height, SMI = (SMM/height ^2^). Finally, to establish the cut-off point of SMI, the specific mean by sex was taken into account <−2 SD to define LMM.

### 2.4 Comparison of cut-off points

A bibliographic search of descriptive studies, cut-off points studies, clinical trials, meta-analysis, and consensus was carried out in electronic databases, including Aminar, Mendeley, Nature, PubMed, Web of Science, Taylor & Francis, Springer, Scopus, and Science Direct. Due to LMM is commonly confused with sarcopenia in the literature, we use both terms for the search. For the search strategy the following MeSH terms and boolean operators were used: (“cut-off points” AND “skeletal muscle mass”) (“cut-off points” AND “sarcopenia”), (“cut-off points” AND “Low skeletal muscle mass”), (“Skeletal muscle mass” AND “bioelectrical impedance analysis”), (“low skeletal muscle mass” AND “elderly”), (“sarcopenia” AND “bioelectrical impedance analysis”),

Studies in English and Spanish with full- text availability recording SMM values estimated by BIA that reported normalized cut-off points of SMI for LMM diagnosis in older adults were included. These cut-off points had to be obtained from the mean value <−2 SD of a young or older reference population differentiated by sex. On the other hand, those studies written in languages other than Spanish and English, were duplicated, defined cut-off points of SMI from appendicular BIA or had measurements with methods other than BIA were excluded.

**Figure 1.**
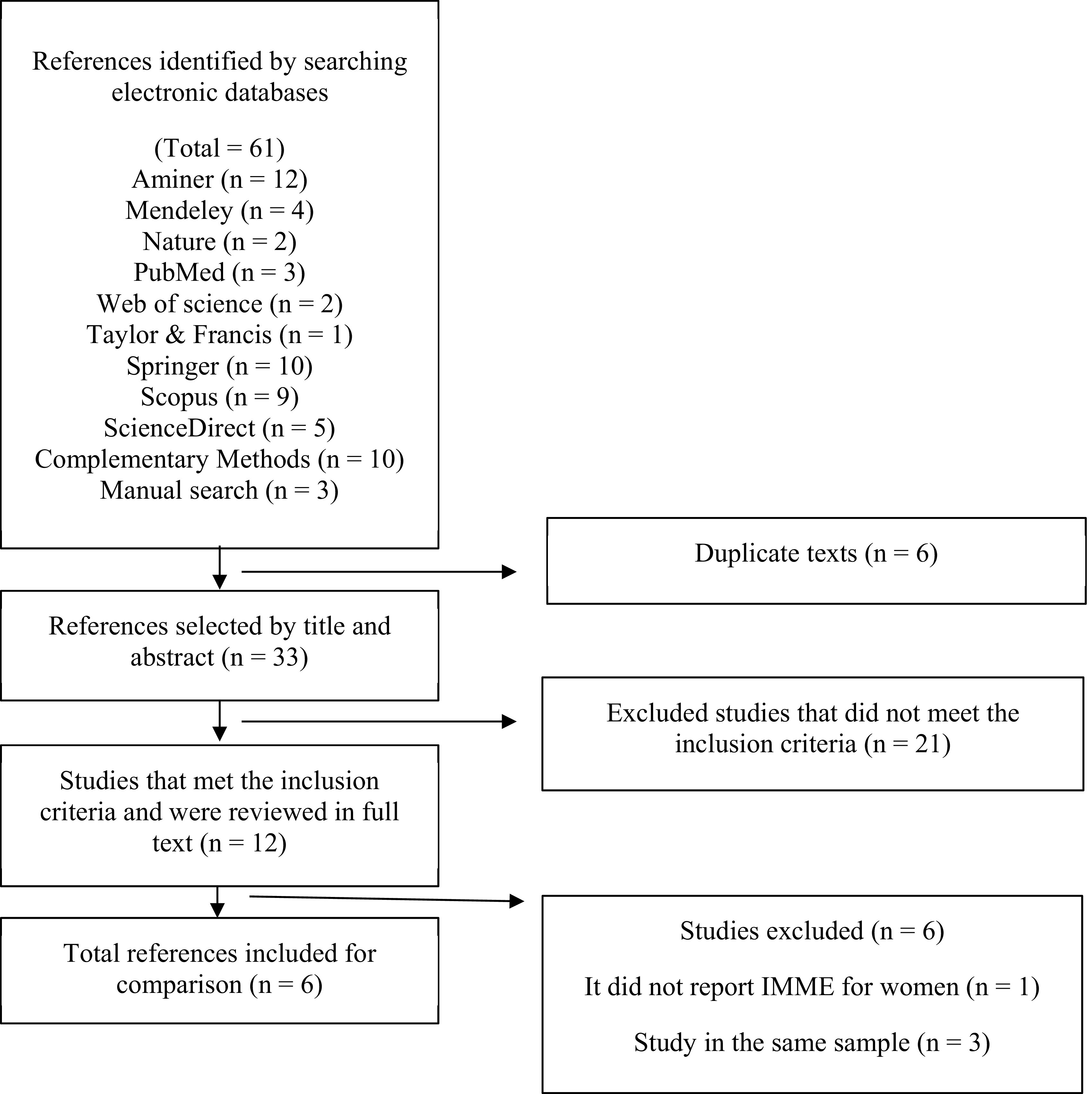
Flowchart on the selection of studies that report cut-off points for SMI for diagnosis of LMM.

The data of the studies found were exported to an Excel spreadsheet (Microsoft Excel 2010; RRID:SCR_016137) to eliminate duplicate references and extract of the purpose of the research, methodological design, population characteristics, description of the BIA device, and recording of the cut-off points for the SMI for each sex ( Figure 1).

### 2.5 Statistical analysis

The Kolmogorov–Smirnov test was used to determine the distribution of the data since more than 50 people were evaluated. For the descriptive analysis of the qualitative variables, absolute values and relative frequencies were used. The quantitative variables were analyzed according to their distribution using means and standard error of the mean. SMI values were classified by sex and were shown as mean and SD of the mean with 95% confidence intervals (CI) for comparison with other studies. Subsequently, the different cut-off points for SMI found in the literature were applied to the 237 older adults in the present study to establish the proportion of people with LMM and identify whether there were significant differences between them. Then the confidence intervals of the difference of these proportions were established. All analyses were carried out with the statistical software SPSS (SPSS version 25; RRID: SCR_002865), licensed by the University of Caldas.

## 3. Results

This study included 237 people older than 60 years. 59.5% were men and in the range of  65 to 69.9 years and constituted 54.4% of the sample. Urban origin was predominated, the right hand was dominant in 225 of the participants and the BMI was higher in women. The SMM for this sample was 27.8 kg for men and 17.3 kg for women. The mean and SD for the SMI were estimated at 9.6±0.8 kg/m ^2^ and 7.5±0.7 kg/m ^2^ for men and women, respectively. In the present study, the cut-off points for SMI were established as −2 SD of the mean of the older adults, then 8.0 kg/m ^2^ for men and 6.1 kg/m ^2^ for women were the final results.

Six articles reported  cut-off points for SMI by BIA. Five of them used tetrapolar technique and measurements at 50 kHz, the remaining four studies do not report this type of data. Most of the studies used the formula of Janssen et al., to estimate the SMI. ^24^ One study reported  SMM  calculation as a multiplication of FFM estimated by BIA with a constant (0.566) and then normalizing it by the height of the subjects. From these six studies, similar to the one reported here, only one established the cut-off points for SMI for a population of healthy adults over 60 years, while the others established it from −2 SD of a young reference group of the same population.

The study by Villada *et al.*, although it was not carried out in the older adults population, did have as its objective the assessment of young adults to determine the cut-off points for LMM in older adults, and that is why it will be included in this comparison, since, additionally, it was carried out in the same geographic region of the present study and we were interested to know whether or not there was a significant difference when applying its cut-off points to our sample.

The range of published cut-off points for SMI for the diagnosis of LMM was 8.25 kg/m ^2^ to 9.31 kg/m ^2^ in men and 5.14 kg/m ^2^ to 7.40 kg/m ^2^ in women. The cut-off points of the SMI of this study and those reported in the literature are presented in Table 1.

**Table 1.** Articles that report SMI cut-off points through BIA.

Author	Population	Team of BIA	Criteria for cut-off point	Cut-off point (kg/m ^2^)
Masanes et al. 2012 ^12^	230 healthy people, 70-80 years old, Spain.	RJL Systems BIA 101	-2SD of Spanish young adults (20-42 years old).	♂8.25; ♀6.68
Han et al. 2016 ^6^	Older adults 878 healthy people,> 65 years, China	MFBIA InBody 720	-2SD of Chinese young adults (20-40 years).	♂7.40; ♀5.14
Bahat et al. 2016 ^8^	406 outpatients,> 60 years, Turkey	Tanita BC-532	-2SD of Turkish young adults (18-39 years old).	♂9.20; ♀7.40
Villada et al. 2018 ^21^	255 healthy young people, 10,835 years old, Colombia	Hydra 4200 Xitron Technologies	-2SD of young Colombian adults (18-35 years old).	♂8.39; ♀6.42
Björkman et al. 2019 ^14^	428 healthy people,> 75 years old, Finland	ImpediMed SFB7	Average value of older adults.	♂9.31; ♀6.90
Bulut et al. 2020 ^19^	1150 healthy people,> 60 years, Turkey	Tanita MC-780U	-2SD of Turkish young adults.	♂8.33; ♀5.70
Pineda et al. 2022 *	237 healthy people,> 60 years old, Colombia	Hydra 4200 Xitron Technologies	-2SD of the mean value for older adults.	♂8.0; ♀6.1

Abbreviations: BIA: Bioelectrical impedance analysis; -2DE: below two standard deviations; ♂: Man; ♀: Woman.

* Present study.

Finally, the cut-off points for the diagnosis of LMM reported in the literature were applied to our sample of 237 healthy older adults from the city of Manizales. It was found that the portion the proportion of people with LMM when applying the different cut-off points ranged from 0.0084 to 0.3544. With the cut-off points of the present study, three persons with LMM were identified, while with the study by Masanes *et al*., 23 cases (CI: 0.0778–0.1162); Han *et al*., zero cases; Bahat *et al*., 84 cases (CI: 0.3233–0.3855); Villada *et al*., four cases (CI: 0.0078–0.0242); Björkman *et al*., 59 cases (CI: 0.2208–0.277) and Bulut *et al*., two cases (CI: 0.0025–0.0143).

Once the confidence intervals of the difference in the proportions were established, a statistically significant difference was found between the LMM results of the cut-off points of the present study and those reported in three of the articles. ^8^^,^^14^^,^^19^ On the other hand, the study by Villada *et al*., did not show a significant difference in the LMM from −2 SD of the mean of older adults or those obtained from −2 SD of the mean in a young population reference from the same region.

## 4. Discussion and conclusion

To the authors’ best knowledge this is the first Latin American study using a sample of healthy older adults living in the community to establish SMI cut-off points for LMM by BIA. In the present study, the cut-off points for this purpose, were <8.0 kg/m ^2^ for men and <6.1 kg/m ^2^ for women. These data are lower than those reported by Bahat *et al*., ^8^ who described 9.20 kg/m ^2^ and 7.40 kg/m ^2^ in men and women, respectively. Perhaps, this difference is due to the fact that the Caucasian population has a different anthropometry than the Latin American ones, as suggested by Gallagher *et al*., ^25^ when they establish that the SMI depends on 80% of the variables height, age, weight, and sex.

SMM is considered an important parameter of body composition, associated with chronic diseases and risk to the health of older adults. ^26^ The BIA technique allows the calculation of the SMI and is valid substitute for total muscle mass since it has a good correlation with the results from the MRI. ^16^^,^^17^ However, the validity and precision of this method depend on the formula and methodology used, so it is important to have an adequate protocol and reference values applicable to the study population. ^27^

When doing the bibliographic review, it was realized that the measurement of the cut-off points of SMI to define LMM has been carried out in few populations around the world, ^28^^,^^29^ so it was not possible to find articles that met the inclusion criteria in Latin American countries. For this reason, and due to the high prevalence LMM, more research is required to obtain baseline data and establish cut-off points for SMI that can be used in clinical practice in order to carry out timely medical interventions. ^12^^,^^15^ Several studies have shown significant differences in the prevalence of LMM when applying cut- off points to populations other than the original population group. ^30^ For this reason, it is suggested that these data obtained for populations of specific ethnic groups. ^31^ Thus, this study represents one of the few that records specific data for a country.

It should be noted that our results showed statistically significant differences when evaluating LMM applying the data obtained in this study and those reported in three studies published in samples from Spain, Turkey and, Finland. ^8^^,^^14^^,^^19^ Thereby, it could be said that cut-off points cannot be used in the populations indistinctly because the genetic characteristics of the populations, anthropometric and occupational differences, type of methodology, equipment, statistical analysis, and cut-off point used. ^32^^,^^33^

This is how that, when applying the cut-off points established in this study, the cases of LMM were significantly lower and differ from those described in other geographical areas. The differences found may be due, not only to the reasons already stated, but also to the fact that most of these studies obtained their data from young reference population and not directly from healthy older adults as in the present study. The cut-off points for SMI were the lowest, perhaps because they were derived from −2 SD from the mean value obtained in a group of healthy older adults and, therefore, the mean value of this population is smaller than the of young adults. ^14^ This is why no universal cut-off points are defined for diagnosing LMM.

It should also be said that in a country with ethnic diversity like Colombia, which also has a different aging index for each region, it is necessary to define regional limits for the application of these cut-off points.

There was no significant difference in the identification of LMM cases applying to our sample the cut-off points found from older or younger adults from the same city (Manizales). The implication in clinical practice is that the use of our cut-off points is more realistic for older adults in our population. By applying cutoff points such as those reported by Bahat *et al.* ^8^ (male: 9.20; female: 7.40), Masanes *et al.* ^12^ (male: 8.25; female: 6.68), or Björkman *et al.* ^14^ (male: 9.31; female: 6.90), they could underestimate or overestimate the prevalence of LLM or sarcopenia in older adults.

The study is not without limitations. The BIA values were not validated by a reference technique such as DXA or MRI and previous studies have reported that the estimation of SMM in older adults using BIA can lead to inaccuracies due to the hydration of their lean mass and the shape of the appendicular muscles. ^34^ However, the technique has been recommended by the EWGSOP. ^31^ Another limitation is that were few patients older than 70 years, and it is recommended to expand the sample from this age in future studies.

On the other said, a strength of this study has to do with the extensive review of the literature to find studies that reported their cut-off points for LMM by BIA. Furthermore, BIA evaluation was carried out under standardized protocols that allowed the results. In addition, the XiTRON Hydra 4200 was used to estimate muscle mass. This is a 3rd generation single-channel tetrapolar BIA device that scans 50 frequencies between 5 kHz and 1 MHz, its accuracy compared to the tedious laboratory dilution methods has been reported numerous times in scientific journals. Scientific studies have also shown the technology to be repeatable and sensitive to small changes, able to detect the volumen distribution differences between subjects. The SMI was calculated from the formula of Janssen *et al.*, ^35^ which was a cross-validated with whole-body MRI a BIA equation, in a sample of 269 Caucasian men and women between 18 and 86 years old. Here it is important to mention that the authors reported this equation as a tool useful for Caucasian, African American and Hispanic populations. Nevertheless, it is recommended to include for future reviews the estimation of LMM from ASMM formula as mentioned by the EWGSOP. In this way, the findings of the present work should be considered under the limitations and strengths expressed.

In conclusion, no significant differences were found when comparing the cut-off points for SMI of population of older and younger adults from the same city, so evaluating LMM from the latter would not cause an overestimation of this pathology. The cut-off points of SMI by BIA derived from a sample of Colombian men and women may be adequate for the diagnosis of LMM in the Colombian geriatric population or populations with similar characteristics to those of the sample evaluated here.

## Data availability

Data are restricted as part of the written informed consent. Data may be obtained for the purposes of research or review from Clara Helena González-Correa ( clara.gonzalez@ucalas.edu.co) on the condition that permission is granted by the participants and for research or review purposes only.
