# Supplementary material for: Cut-off points for low skeletal muscle mass in older adults: Colombia versus other populations
Source: F1000Res. 2023 Jun 27;11:304. Originally published 2022 Mar 11. [Version 2] doi: 10.12688/f1000research.109195.2 (PMC10450257; doi:10.12688/f1000research.109195.2)
Supplement: Peer review history

# Peer review: Reviewer response for version 2

**DOI:** 10.5256/f1000research.151624.r191603

**Published:** 2023-08-24

**Authors:** Idil Yavuz

**Affiliations:** 1 Department of Statistics, Faculty of Science, Dokuz Eylul University, Izmir, Turkey

Summary:

 This prospective study aims to provide cut-off points for SMI for the Colombian geriatric population in order to aid diagnosis of sarcopenia. The authors also provide a review on existing studies and compare their own findings with the literature. The study provides valuable information on this subject especially for the Latin American population however I have a few concerns as detailed below.

 Comments:

 1. The manuscript needs to be edited for grammar.

 2. In Results 1st paragraph, the authors say:

 "BMI was higher in women". Was any statistical comparison performed? If so details need to be provided..

 2. In Results last paragraph, the authors say:

 "Once the confidence intervals of the difference in the proportions were established, a statistically significant difference was found between the LMM results of the cut-off points of the present study and those reported in three of the articles."

 How were the proportions compared? Did the authors carry out pairwise comparisons as they only mention that the CI for differences were calculated? A more appropriate analysis would be to test for the overall effect of country on the LLM variable. Statistical approach here needs to be clarified and possibly corrected.

 3. In Discussion the authors say:

 "There was no significant difference in the identification of LMM cases applying to our sample the cut-off points found from older or younger adults from the same city (Manizales)."

 This sentence is not clear and the statement needs statistical evidence.

 4. The manuscript lacks summary statistics, summary tables for statistical findings etc. More numerical results like p-values, types of statistical tests applied, significance levels etc. need to be included in the write up. 

Is the work clearly and accurately presented and does it cite the current literature?

Yes

If applicable, is the statistical analysis and its interpretation appropriate?

Partly

Are all the source data underlying the results available to ensure full reproducibility?

Yes

Is the study design appropriate and is the work technically sound?

Yes

Are the conclusions drawn adequately supported by the results?

Partly

Are sufficient details of methods and analysis provided to allow replication by others?

Partly

Reviewer Expertise:

Statistics, Biostatistics

I confirm that I have read this submission and believe that I have an appropriate level of expertise to confirm that it is of an acceptable scientific standard, however I have significant reservations, as outlined above.

# Peer review: Reviewer response for version 1

**DOI:** 10.5256/f1000research.120671.r164396

**Published:** 2023-05-11

**Authors:** Miriam T. López-Teros

**Affiliations:** 1 Medical, Dental and Health Sciences Program, National Autonomous University of Mexico, Mexico City, Mexico

The objective of this article is to define cut-off points for LMM (low muscle mass) using SMI (skeletal muscle mass index) evaluated by BIA (bioimpedance) for Colombian older adults, which can be used for the diagnosis of sarcopenia. It is pertinent since the importance of having specific cut-off points for each region is recognized, in addition to the fact that there are few published studies on SMM (Whole-body skeletal muscle mass) or ASMM (appendicular skeletal muscle mass) cut-off points. The importance of having specific cut-off points for each region to define LMM is recognized.

**Observations:**

Introduction:

- It is suggested to mention the difference between obtaining Whole-body skeletal muscle mass (SMM) and not ASMM to define LMM, as mentioned in EWGSOP2.
- I don't know if in this analysis the ASMM could have been better calculated, this formula could have been used for its estimation that is mentioned in the EWGSOP2 (Yamada *et al.*, 2017 ^1^).

Methodology:

- In relation to the previous comment, the MesH term "appendicular skeletal muscle mass" could have been added to the systematic review.

Statistic analysis:

- It is mentioned that the proportion of subjects with LMM in the present study was compared with the results reported in the studies included in the review, but it is not mentioned what statistical tests were used for this comparison.

Results:

- It is recommended to add a table of the general characteristics of the study population, such as means and frequencies of variables such as age, sex, comorbidity, anthropometric measurements (weight, height, BMI), SMI, among others.
- It is mentioned that there were significant differences between the proportion of subjects with LMM in the present study versus studies reported in the review, it is suggested to add the p value or the 95% CI found in the statistical tests.

Discussion:

- To go deeper into using SMM and not ASMM to define LMM, as well as about the model used Hydra 4200 Xitron Technologies bioimpedancemeter®, which has previously been reported on the accuracy of this equipment.

Is the work clearly and accurately presented and does it cite the current literature?

Yes

If applicable, is the statistical analysis and its interpretation appropriate?

Partly

Are all the source data underlying the results available to ensure full reproducibility?

No source data required

Is the study design appropriate and is the work technically sound?

Yes

Are the conclusions drawn adequately supported by the results?

Yes

Are sufficient details of methods and analysis provided to allow replication by others?

Yes

Reviewer Expertise:

Nutrition and geriatric, with researc topics: sarcopenia, frailty, physical performance,  functional dependence, dietary patterns and food security in older adults.

I confirm that I have read this submission and believe that I have an appropriate level of expertise to confirm that it is of an acceptable scientific standard.

## References

[1] : Developing and Validating an Age-Independent Equation Using Multi-Frequency Bioelectrical Impedance Analysis for Estimation of Appendicular Skeletal Muscle Mass and Establishing a Cutoff for Sarcopenia. *Int J Environ Res Public Health* .2017;14(7) : 10.3390/ijerph14070809 10.3390/ijerph14070809 28753945PMC5551247

# Response

**Published:** 2023-06-14

**Authors:** Clara Helena González-Correa

**Affiliations:** Universidad de Caldas, Colombia

**Dear reviewer,**

 We welcome your comments and suggestions about our work. We will take into account what you suggest to improve the academic merit of our research.

**1. It is suggested to mention the difference between obtaining Whole-body skeletal muscle mass (SMM) and not ASMM to define LMM, as mentioned in EWGSOP2.**

 The reason why we use total and not appendicular SMI, is because in the investigations carried out in our research laboratory a protocol has been used since 2013 to standardize the measurement of patients (Bioelectrical impedance analysis (BIA): A proposal for standardization of the classical method in adults. 2012 J. Phys.: Conf. Ser. 407 012018). 

 Nevertheless, we are very grateful for your suggestion, as this is an aspect that we would like to take into account from now on in our research, taking into account the importance of evaluating LMM from the formula for ASMM as mentioned in EWGSOP2. 

**2. It is mentioned that the proportion of subjects with LMM in the present study was compared with the results reported in the studies included in the review, but it is not mentioned what statistical tests were used for this comparison.**

 In the statistical analysis section, it was described: "SMI values were classified by sex and were shown as mean and SD of the mean with 95% confidence intervals for comparison with other studies. Subsequently, the different cut-off points for SMI found in the literature were applied to the 237 older adults in the present study to establish the proportion of people with LMM and identify whether there were significant differences between them. Then the confidence intervals of the difference of these proportions were established". 

**3. It is recommended to add a table of the general characteristics of the study population, such as means and frequencies of variables such as age, sex, comorbidity, anthropometric measurements (weight, height, BMI), and SMI, among others.**

 In this case, as the general characteristics of the participants were few, we described them in text form in the initial part of the results. Thus, we describe the mean and standard deviation of age, gender, origin, and predominant dominance. We also mentioned that the BMI was higher in the women included in the study. We also described the mean and standard deviation of SMM and SMI by gender. Thank you very much for this recommendation, it will be taken into account for future research. 

**4. It is mentioned that there were significant differences between the proportion of subjects with LMM in the present study versus studies reported in the review, it is suggested to add the p-value or the 95% CI found in the statistical tests.**

 ICs were added to the results session: "Finally, the cut-off points for the diagnosis of LMM reported in the literature were applied to our sample of 237 healthy older adults from the city of Manizales. It was found that the portion the proportion of people with LMM when applying the different cut-off points ranged from 0.0084 to 0.3544. With the cut-off points of the present study, three persons with LMM were identified, while with the study by Masanes et al., 23 cases (CI: 0,0778- 0,1162); Han et al., zero cases; Bahat et al., 84 cases (CI: 0,3233-0,3855); Villada et al., four cases (CI: 0,0078-0,0242); Björkman et al., 59 cases (CI: 0,2208-0,277) and Bulut et al., two cases (CI: 0,0025-0,0143)". 

**5. To go deeper into using SMM and not ASMM to define LMM, as well as about the model used Hydra 4200 Xitron Technologies bioimpedancemeter®, which has previously been reported on the accuracy of this equipment.**

 It was included in the discussion: 

 "In addition, the XiTRON Hydra 4200 was used to estimate muscle mass. This is a 3rd generation single-channel tetrapolar BIA device that scans 50 frequencies between 5kHz and 1MHz, its accuracy compared to the tedious laboratory dilution methods has been reported numerous times in scientific journals. Scientific studies have also shown the technology to be repeatable and sensitive to small changes, able to detect the volume distribution differences between subjects".

# Peer review: Reviewer response for version 1

**DOI:** 10.5256/f1000research.120671.r147195

**Published:** 2022-08-30

**Authors:** Gulistan Bahat, Asli Tufan

**Affiliations:** 1 Division of Geriatrics, Department of Internal Medicine, Istanbul Medical School, Istanbul University, Istanbul, Turkey; 2 Medical Faculty, Marmara University, Istanbul, Turkey

This is a prospective study aiming to define local cut-off points for low muscle mass and to compare them with the existing cut-off points in the literature. A “one size fits all” approach can be misleading in the diagnosis of sarcopenia. EWGSOP has therefore recommended that national cut-off points be used for sarcopenia diagnosis. This study fills a gap in the definition of sarcopenia and is of interest to the readership of the journal. However, some major and minor points need to be clarified.

**Major point:**

 For sarcopenia diagnosis, EWGSOP recommends using the normative data of healthy young adults. Why have the authors used the normative data of older adults for LMM diagnosis?

**Minor points:**

 “237 elderly patients from the city of Manizales who live in the community were included in the study. The patients were evaluated in the University of Caldas health service provider.” It is not quite clear how the patients were recruited. Were they hospital outpatients? If so, they are expected to be relatively more disabled compared to the older population in general, which may be considered as a limitation of the study.

 The abbreviations under table 1 reads: 

 -2 **DE**: below two standard deviations, which should have been “-2 **SD**” (also in the table: “-2DE of Turkish young adults”).

 It would be better if the discussion started with “To the authors’ best knowledge…”

 There are minor grammatical and semantic errors throughout the text.

 “The implication in the clinical practice is that the use of our cut-off points from a young population in closer to real data form elders and prevents underestimation or overestimation of the prevalence of LLM or sarcopenia in older adults as if it could happen if, for example, the cut-off points reported by Bahat *et al*. ^8^ (male: 9.20; female: 7.40) were used for our population, or, even more serious, if the references by a Masanes et al.12 (male: 8.25; female: 6.68), or Björkman *et al*. ^14^ (male: 9.31; female: 6.90) were applied.” This sentence in the discussion section is too long and confusing.

 In order to avoid ageism, the term “elderly” should be replaced with “older adults”.

Is the work clearly and accurately presented and does it cite the current literature?

Yes

If applicable, is the statistical analysis and its interpretation appropriate?

Yes

Are all the source data underlying the results available to ensure full reproducibility?

Yes

Is the study design appropriate and is the work technically sound?

Yes

Are the conclusions drawn adequately supported by the results?

Yes

Are sufficient details of methods and analysis provided to allow replication by others?

Yes

Reviewer Expertise:

sarcopenia

We confirm that we have read this submission and believe that we have an appropriate level of expertise to confirm that it is of an acceptable scientific standard, however we have significant reservations, as outlined above.

# Response

**Published:** 2022-08-30

**Authors:** Clara Helena González-Correa

**Affiliations:** Universidad de Caldas, Colombia

*Dear reviewer,*

 We welcome your comments and suggestions about our work. We will take into account what you suggest to improve the academic merit of our research.

 We will now respond to the first two comments. The other observations regarding terminology and grammatical errors will be taken into account and modified in the paper once the F1000 editorial authorizes it.

***For sarcopenia diagnosis, EWGSOP recommends using the normative data of healthy young adults. Why have the authors used the normative data of older adults for LMM diagnosis?***

 Although EWGSOP recommends using the normative data of healthy young adults, we used the

 normative data directly from older adults for LMM diagnosis because we consider that the rapidly

 changing dynamics of lifestyles and morbidities can create huge differences between a young

 generation from which reference values are taken and an older adult generation that may have a

 difference of 50 or more years. We wanted to see whether or not the cut-off points for low muscle

 mass resulting from the young were similar to those obtained from a population of older adults, all

 at ages corresponding to the same generation.

***If so, they are expected to be relatively more disabled compared to the older population in general, which may be considered as a limitation of the study.***

 The inclusion criteria considered being over 60 years of age, being a resident of Manizales, not having a sarcopenia diagnosis, and being independent in carrying out activities of daily living.

 The University of Caldas health service provider serves people not only from the University but is

 open to the entire community of the city. The volunteers were tested there but were recruited

 among independent people living in the community, who did not have pathologies that would

 affect the measurements of muscle mass and were considered healthy. Wide dissemination was

 carried out through social networks, word of mouth, pamphlets, the University's website, and

 other radio news media in the city.
